# Negative BOLD fMRI Response in the Visual Cortex Carries Precise Stimulus-Specific Information

**DOI:** 10.1371/journal.pone.0000410

**Published:** 2007-05-02

**Authors:** David Bressler, Nicole Spotswood, David Whitney

**Affiliations:** The Department of Psychology and The Center for Mind and Brain, University of California Davis, Davis, California, United States of America; Claremont Graduate University, United States of America

## Abstract

Sustained positive BOLD (blood oxygen level-dependent) activity is employed extensively in functional magnetic resonance imaging (fMRI) studies as evidence for task or stimulus-specific neural responses. However, the presence of sustained negative BOLD activity (i.e., sustained responses that are lower than the fixation baseline) has remained more difficult to interpret. Some studies suggest that it results from local “blood stealing” wherein blood is diverted to neurally active regions without a concomitant change of neural activity in the negative BOLD regions. However, other evidence suggests that negative BOLD is a result of local neural suppression. In both cases, regions of negative BOLD response are usually interpreted as carrying relatively little, if any, stimulus-specific information (hence the predominant reliance on positive BOLD activity in fMRI). Here we show that the negative BOLD response resulting from visual stimulation can carry high information content that is stimulus-specific. Using a general linear model (GLM), we contrasted standard flickering stimuli to a fixation baseline and found regions of the visual cortex that displayed a sustained negative BOLD response, consistent with several previous studies. Within these negative BOLD regions, we compared patterns of fMRI activity generated by flickering Gabors that were systematically shifted in position. As the Gabors were shifted further from each other, the correlation in the spatial pattern of activity across a population of voxels (such as the population of V1 voxels that displayed a negative BOLD response) decreased significantly. Despite the fact that the BOLD signal was significantly negative (lower than fixation baseline), these regions were able to discriminate objects separated by less than 0.5 deg (at ∼10 deg eccentricity). The results suggest that meaningful, stimulus-specific processing occurs even in regions that display a strong negative BOLD response.

## Introduction

In functional MRI (fMRI) experiments, the neural response to visual stimulation is inferred from the change in the BOLD (blood oxygenation level-dependent) signal. In a typical experiment, subjects view a stimulus in the test condition and a blank screen in the control condition. When these two conditions are contrasted in data analysis, researchers attribute perceptual and cognitive functions to regions that display a positive BOLD response compared to baseline, since positive BOLD is closely coupled with neuronal activity [1], [2]. However, researchers routinely discount regions that display a negative BOLD response (a lower level of BOLD response for the stimulus than for the blank screen), because negative BOLD has proven to be much harder to characterize. In this paper, we show that patterns of negative BOLD activity carry meaningful information about stimulus-specific visual processing. Therefore, to understand the neural correlates of visual perception, fMRI studies should consider the negative BOLD signal as informative.

Much of the debate over the negative BOLD signal has centered on whether its source is primarily vascular or neuronal. However, neither explanation of the *origin* of negative BOLD makes strong claims about its *meaning*. In fact, most research on the negative BOLD signal downplays its relevance to the task or stimulus. Numerous studies have characterized the negative BOLD signal as stimulus-independent, spatially widespread and diffuse, and varying little in position across a variety of tasks [3], [4], [5], [6]. Although a few show some change in the pattern of negative BOLD activity when visual stimulation changes, it is not clear to what extent these changes are precise [7], [8], [9]. In this study we use a novel technique to show that patterns of negative BOLD responses, far from being unrelated to the stimulus, are highly informative and stimulus-specific.

Our goal was to determine the spatial selectivity of negative BOLD responses. To do this, we presented stimuli in slightly different positions, each of which produced a unique pattern of positive and negative BOLD activity. Within the regions that selectively displayed a negative BOLD response, we cross-correlated patterns of fMRI activity produced by each stimulus. If the information carried in the negative BOLD response is not spatially precise, then there should be no difference in the pattern of negative BOLD activity for objects in slightly different positions. Our results will show that, on the contrary, as the stimuli were shifted further from each other, the correlation in the spatial pattern of activity decreased significantly. The results suggest that meaningful, stimulus-specific processing occurs even in regions that display a strong negative BOLD response.

## Results

**Figure 1:**
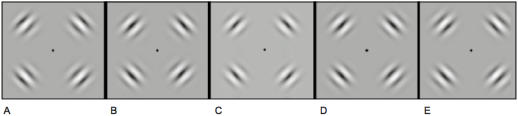
Stimuli used in the experiment. A–E. Four flickering Gabors were presented at one of five eccentricities; the standard deviation of each Gabor's contrast envelope was incrementally skewed by ∼0.19 deg toward (A–B) or away (D–E) from the fovea. The Gabors in (E) are skewed away from the Gabors in (A) by 0.77 deg (see Materials and Methods).

**Figure 2:**
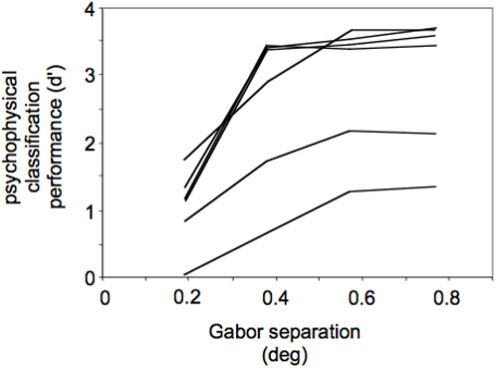
Psychophysical results for six subjects. While in the scanner, subjects reported which condition they were viewing on each trial (5AFC classification task). The abscissa on the graph shows the difference in the eccentricity between any two of the five conditions (e.g., the difference in skew between Fig. 1A and 1B was 0.19 deg, while the difference between Fig. 1A and 1E was 0.77 deg). The ordinate shows discrimination (d-prime, calculated from the 5AFC classification data [54]). When two stimulus conditions were similar (e.g., Fig. 1A and 1B), subjects had difficulty classifying which condition they were viewing, resulting in a lower d-prime. Conditions that were separated by greater eccentricities yielded higher discrimination ability. For all subjects, discrimination improved with an increasing difference in the skew of the envelope. Overall ability varied between subjects, but the trend was consistent across subjects, and was significant for each subject (least significant log fit to the data was for subject MC, F_(1,18)_ = 9.67, P<0.01).

The stimuli were four flickering Gabors arranged in one of five locations (see Materials and Methods; Fig. 1). The eccentricity of the peak contrast was identical across all 5 Gabor arrangements (9.05 degrees), but the standard deviation of the contrast envelope was skewed either toward or away from the fovea (by 0.38, 0.19, 0, −0.19, or −0.38 deg relative to the symmetrical Gabor at 9.05 deg; Fig. 1). On each trial, subjects reported the magnitude of the Gabor skew (5-alternative forced choice classification task, similar to the method of single stimuli; [10]). Figure 2 shows the psychophysical results for each subject. With increasing separation between the flickering Gabors, subjects were better able to accurately classify the stimuli (e.g., discriminating between Fig. 1A and 1E is relatively easy). The overall ability varied between subjects, but the trend was consistent across subjects and significant for each subject (least significant log fit to the data was for subject MC, F_(1,18)_ = 9.67, P<0.01). Six of the seven subjects were able to discriminate Gabors separated by 0.19 deg in eccentricity, and all seven subjects were able to discriminate differences of 0.77 deg eccentricity (lowest d′ was 1.2 for subject MC). The precision with which subjects judged Gabor locations is consistent with several previous studies on acuity in the periphery [11], [12], [13], even in absence of references [14]. Further, these psychophysical data confirm previous reports that the method of single stimuli can be used to measure acuity even though subjects must rely on an implicit standard when judging each stimulus [10], [15], [16].

**Figure 3:**
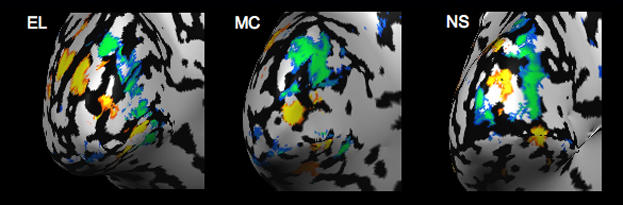
Cortical surface maps for three representative subjects showing regions of positive (yellow-red) and negative (blue-green) BOLD activity. The maps were generated by fitting a general linear model to the data and contrasting all of the flickering Gabor stimuli (Fig. 1) to a fixation baseline; the threshold for these maps was set at t = 5.6, P_(Bonf)_<0.001.

**Figure 4:**
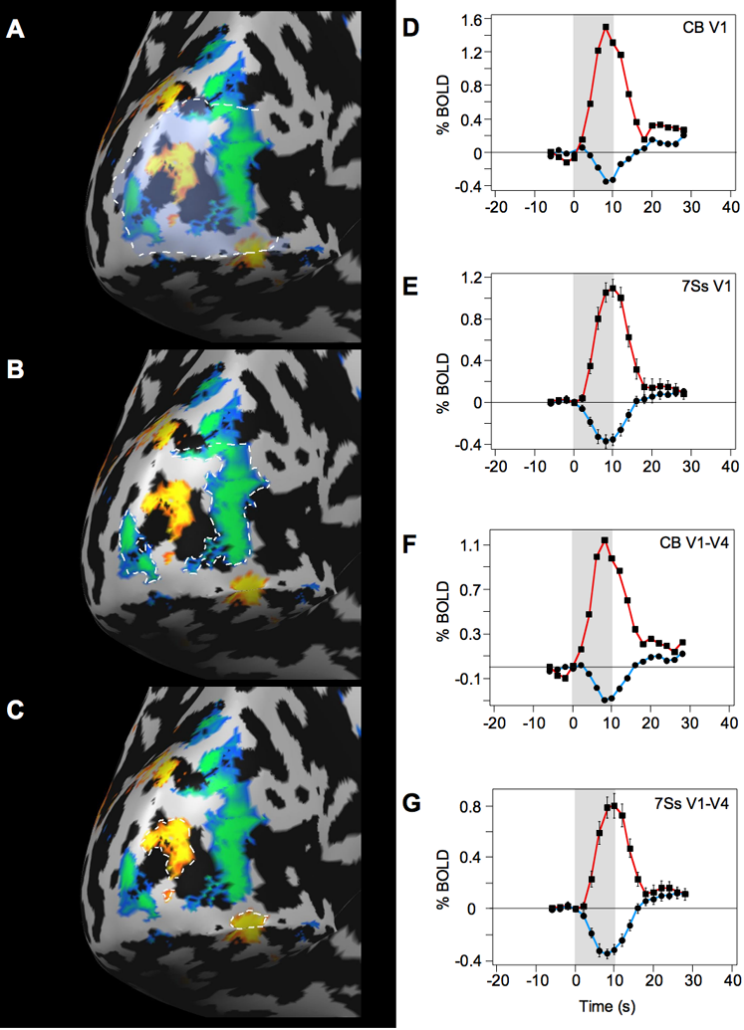
Surface map for representative subject and time course of BOLD response for all subjects. A. Cortical surface for one subject showing visual area V1 (outlined), measured in separate retinotopic mapping runs (see Materials and Methods). B. Negative BOLD ROI in V1 for the representative subject. C. Positive BOLD ROI in V1 for the representative subject. D–E. Event-related average timecourses were measured separately for positive and negative BOLD ROIs. Positive BOLD ROI (red line, squares) and the negative BOLD ROI (blue line, circles) for a representative subject (D) and for the group of subjects (E). F–G. Positive and negative BOLD ROI responses averaged across visual areas V1, V2, V3, V3A, VP, and V4 for one representative subject (F), and for the group of subjects (G). The gray filled region in each graph shows the stimulus presentation (10 s). Error bars, ±s.e.m.

In the fMRI analysis, we contrasted the flickering Gabors with the fixation baseline, which produced significant positive and negative BOLD activity throughout the visual cortex (Fig. 3). Consistent with previous studies [5], [7], the negative BOLD response surrounded the positive BOLD response in a manner reminiscent of a Mexican-hat or difference-of-Gaussian operator. For each subject, we selected separate non-overlapping regions of interest (ROIs) of significant positive and negative BOLD activity in visual area V1 (blue and orange activity circled with white dashed lines, respectively, in Fig. 4B–C; see Materials and Methods). The negative BOLD ROI was defined, for each subject, as the union of all regions within V1 that displayed a significant negative BOLD response; the positive BOLD ROI was defined similarly. Figure 4 shows the corresponding BOLD time courses in the negative and positive BOLD ROIs for one representative subject and for the group of seven subjects. In V1, the peak negative response in the negative BOLD ROIs was −0.37% (t_(6)_ = 6.0, P = 0.001), and the peak positive response in the positive BOLD ROIs was +1.1% (t_(6)_ = 13.4, P<0.001). The results demonstrate that flickering stimuli generate not only positive responses but significant negative responses as well, confirming several previous studies [5], [6], [9], [17], [18].

**Figure 5:**
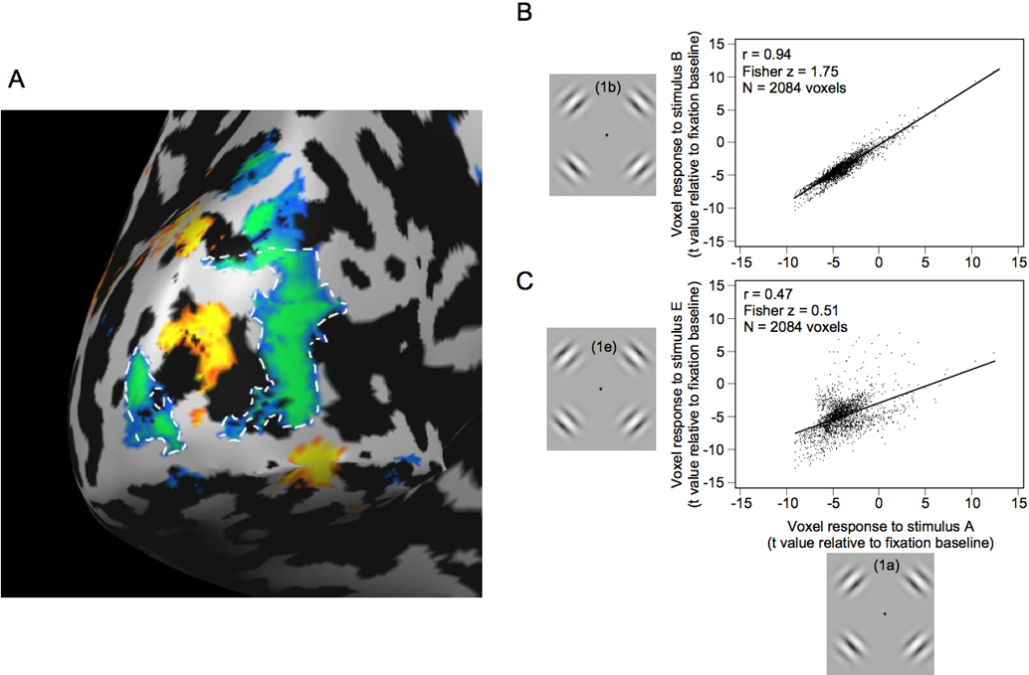
Measuring position discrimination in the visual cortex. A. A representative subject's negative BOLD ROI (circled with dashed white line), composed of 2084 voxels. B. The response of each of the 2084 voxels in the negative BOLD ROI is plotted for two of the conditions (Fig. 1A and 1B). The abscissa shows the response to the stimulus in Fig. 1A (t score generated by a general linear model contrast relative to fixation baseline, see Materials and Methods). The ordinate shows the response to the stimulus in Fig. 1B. Across the population of voxels, there was a strong correlation in the responses to the two stimuli (r = 0.94, P<0.001). This is not surprising, given how similar the two conditions were. C. Within the same ROI, the response to the stimulus in Fig. 1A was compared to the response to the stimulus in Fig. 1E (a condition in which the Gabors were positionally skewed by 0.77 deg). Across the population of 2084 voxels, the correlation was r = 0.47. There was a significantly stronger correlation in (B) than in (C) (Fisher z difference = 1.75−0.51 = 1.24, Z = 44.2, P<0.001). That is, the Gabors that were close to each other produced a higher correlation than the Gabors that were separated by a greater distance.

Within each subject's negative BOLD ROI in V1, we cross-correlated the patterns of activity produced by each of the five different stimulus conditions (see Materials and Methods and Fig. 1), which produced a total of ten correlation coefficients for each subject. Each correlation reflects the similarity in the pattern of activity for a particular pair of conditions (i.e., Gabor positions). For example, within each subject's negative BOLD ROI in V1 (Fig. 5a), the correlation between the pattern of activity for Gabor stimuli that were close to each other (Fig 5b) was much higher than the correlation between the pattern of activity for two Gabor locations that were farther apart (Fig 5c). We converted the correlation coefficients to Fisher z-scores because these are linear and can be directly compared [19]. If a given population of voxels (an ROI) is sensitive to object position, we expect that the spatial correlation in the responses across that ROI will decrease as the distance between Gabor locations increases.

**Figure 6:**
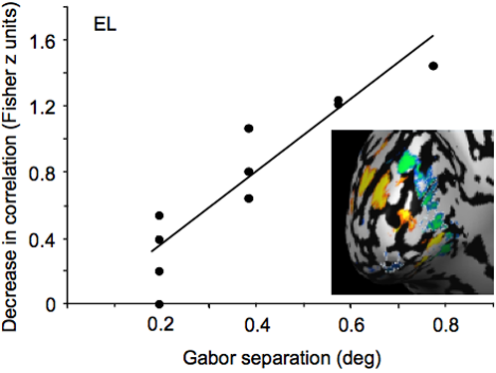
Position discrimination within the negative BOLD ROI of one representative subject's visual area V1. Within the negative BOLD ROI (circled in white dashed line), the pattern of responses to each of the five stimulus conditions (Fig. 1) were cross-correlated (the analysis from Fig. 5 was repeated for every pair of stimulus conditions). All correlations were converted to Fisher z scores and normalized to (subtracted from) the highest correlation (ordinate). Zero on the ordinate therefore indicates a high spatial correlation. The abscissa shows the difference in the eccentricity of any pair of conditions (ranging from 0.19 deg to 0.77 deg, as in Fig. 1). The graph indicates that as the eccentricity of the Gabor conditions is increasingly separated, the correlation across the spatial pattern of activity decreased (indicated by a positive slope in the data). A linear regression revealed a significant effect of Gabor separation on the spatial correlation (*f(x)* = 2.2*x*−0.08; F_(1,8)_ = 43.6, P<0.001).

Figure 6 shows an analysis of the negative BOLD ROI of a representative subject's visual area V1. As the positions of the Gabor stimuli were separated (abscissa), there was a decrease in the spatial correlation of activity across the ROI (indicated by the positive slope in the data; note that increasing values along the ordinate of the graph indicate a decrease in the spatial correlation). The slope of the linear regression is a measure of the ROI's sensitivity to minute shifts in the position of the Gabors; a higher slope reflects greater selectivity to object position. Notice that if a region demonstrated no selectivity for stimulus position, the slope would be zero, because there would be no difference between the spatial correlation of activity for nearby object positions and the correlation for more distant positions. However, if a region is highly selective for object position, there would be a high correlation for nearby positions but a low correlation for more distant positions, producing a steep position discrimination slope. A linear regression of the data in Figure 6 revealed a significant slope of 2.2 (F_(1,8)_ = 43.6, P<0.001), which indicates that skewing the positions of the Gabors by 1 deg visual angle would produce a reduction in the spatial correlation of 2.2 Fisher z units (0.976 r units).

**Figure 7:**
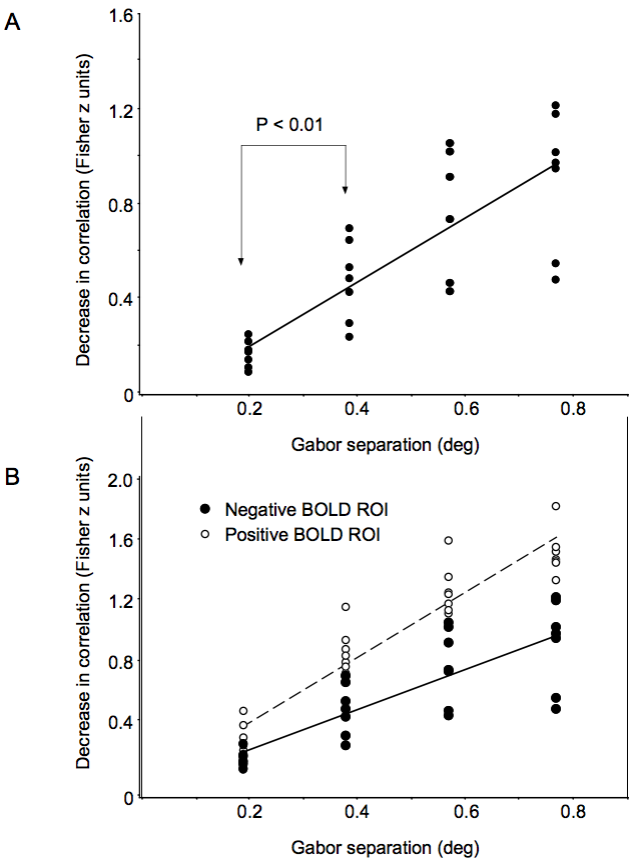
Position discrimination in V1. A. Position discrimination in the negative BOLD ROI across all seven subjects. A linear regression revealed a significantly positive position discrimination slope (slope of 1.34; F_(1,26)_ = 52.9, p<0.01). B. Within each subject's V1, the same analysis was applied to the positive BOLD ROIs (open circles). The slope of the position discrimination function in the positive BOLD ROI in V1 was 2.15 (F_(1,26)_ = 202.1, P<0.001). Although the regions that display a positive BOLD response are better able to discriminate object position (F_(1,6)_ = 49.3, P<0.01), the regions that display a negative BOLD response are still able to discriminate object position with remarkable precision.

Figure 7A shows the position discrimination slope in the negative BOLD V1 ROIs for all seven subjects. A linear regression revealed a significantly positive slope of 1.34 (F_(1,26)_ = 52.9, P<0.01). This indicates that shifting the Gabor stimuli by one degree of visual angle resulted in a decrease in the spatial correlation across the population of voxels of 1.34 Fisher z units. This is equivalent to a decrease of 0.87 Pearson r units—a dramatic decorrelation in the spatial pattern of activity, despite a tiny change in the position of the objects. An analysis of variance (ANOVA) confirmed a significant effect of Gabor position (F_(3,24)_ = 17.9, P<0.01). Finally, the difference in Fisher z-scores (spatial correlations) between the smallest Gabor separation (0.19 vs. 0.38 deg) was significant (t_(6)_ = 8.0, P<0.01). The fact that the slope was significantly positive, with very little variation, shows that this method is able to reveal position coding on an extraordinarily precise scale.

Although the regions that display a negative BOLD response clearly have patterns of activity that are capable of discriminating object position (Fig. 7A), how does this compare to regions that display a positive BOLD response? To address this, we repeated the analysis on the regions of significant positive BOLD in V1. As expected, subjects' positive BOLD activity was also highly sensitive to shifts in stimulus position (Fig. 7B). A linear regression on the within-subjects averaged z-scores revealed a significantly positive slope of 2.15 (F_(1,26)_ = 202.1, P<0.001). Position discrimination in the positive ROIs in V1 was significantly better than in the negative ROIs (F_(1,6)_ = 49.3, P<0.01).

**Figure 8:**
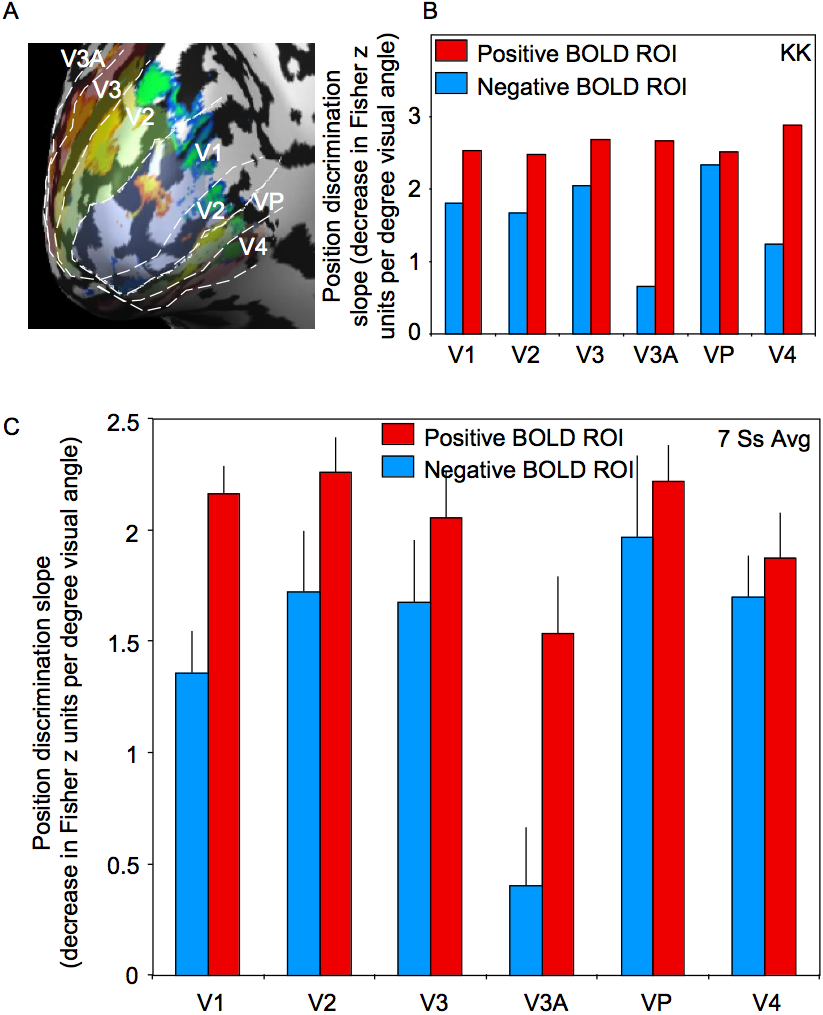
Position discrimination across visual areas V1, V2, V3, V3A, VP, and V4. A. Representative cortical surface map for one subject. B. The position discrimination slope for the negative (blue) and positive (red) BOLD ROIs within each visual area for the representative subject shown in (A). The position discrimination slope for each ROI was calculated as in Fig. 6. C. The position discrimination slope averaged across all seven subjects. For the positive BOLD ROIs (red bars), there was a significantly positive position discrimination slope across visual areas V1 through V4, indicating that all of these visual areas are topographically precise (i.e., they can detect 0.19 deg shifts in the position of an object at 9 deg eccentricity). The same was true for the negative BOLD ROIs as well. Of all the areas tested here, the least significant position discrimination was in the negative BOLD ROI in V3A (t_(5)_ = 3.2, P<0.05). Error bars, ±1 s.e.m.

The results above show that regions of V1 that display a negative (and a positive) BOLD response to a particular object contain patterns of responses that are highly selective for the object's position; objects in slightly shifted positions produce increasingly different patterns of activity. To determine if the precise discrimination of object position was restricted to V1, we repeated the analysis above separately in visual areas V1 through V4. Figure 8A shows a representative subject's positive and negative BOLD activity on an inflated cortical surface, showing visual areas V1 through V4. Within each visual area, we selected separate ROIs of significant positive and negative BOLD and performed the linear regression analysis (as in Fig. 7). For the representative subject (Fig. 8B), and for all seven subjects together (Fig. 8C), there was a significantly positive position discrimination slope across all visual areas V1–V4 for both positive and negative BOLD ROIs. The least significant position discrimination was in the negative BOLD ROI in area V3A (t_(5)_ = 3.2, P<0.05). Interestingly, the patterns of negative and positive BOLD responses in visual areas V2, V3, VP, and V4 discriminated minute shifts in object position about as well as V1 (there was no significant difference in the position discrimination slope across these visual areas). Overall, the positive BOLD ROIs were better able to discriminate object position (F_(1,5)_ = 9.64, P<0.05). Nevertheless, the regions that displayed a negative BOLD response discriminated object position with great precision. Therefore, the pattern of negative responses carries meaningful and precise information about object position.

The position discrimination slopes within both positive and negative BOLD regions were comparable to the psychophysical results in Figure 2. Subjects were able to correctly classify objects separated by greater than 0.19 deg, and the slope of the position discrimination functions in Figures 7 and 8 indicates that the pattern of positive and negative BOLD activity was also able to discriminate objects shifted by this amount. The consistency between the psychophysical and the BOLD data indicates that our statistical technique is not limited by fMRI methodology or the coarse resolution of BOLD sampling. Future studies could extend this technique to reveal the physiological mechanisms of other fine scale processes.

**Figure 9:**
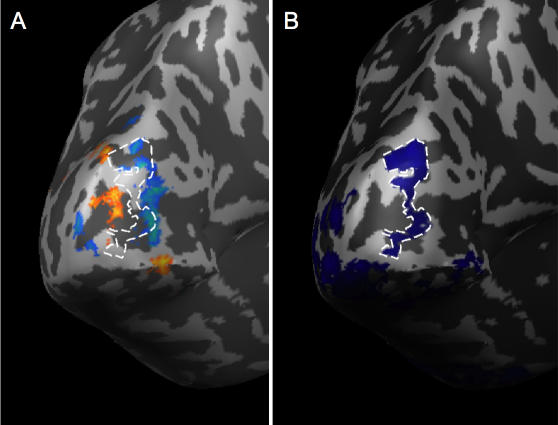
Regions of the visual cortex that were most sensitive to stimulus position. A. The positive (red-orange) and negative (green-blue) BOLD response to the flickering Gabors for a representative subject. B. Position discrimination slopes (as in Fig. 7) were measured for every possible 5 mm^3^ ROI in the occipital lobe (see Materials and methods). Those overlapping ROIs that showed the steepest position discrimination slopes are shown in dark blue and outlined with a dashed white line. Notice that the region of the visual cortex that is most sensitive to stimulus position (within the white dashed line) falls between the positive and negative BOLD regions in (A). This supports the idea that the edges of the object representation, where the BOLD response changes from positive to negative, are especially important for object localization [37], [48].

The pattern of activity in each ROI we tested above displayed extremely precise selectivity for object position. However, if the activity in V1 produced by an object is retinotopically localized, then we would only expect precise discrimination of that object's position to be possible in regions near the retinotopic representation of that object. Therefore, we should only find our steep position discrimination functions (like Figs. 7 and 8) in select locations, not everywhere in the visual cortex. Moreover, if the negative BOLD response to an object is capable of coding that object's position, it should be directly related to the retinotopically precise positive BOLD response to the object [7]. To address this, we separately defined every possible 5×5×5 cube of voxels in the entire occipital lobe (creating thousands of ROIs), performed the analysis above to obtain a position discrimination slope value for each ROI, and then created a new surface map that shows clusters of voxels that are best able to discriminate object position (see Materials and Methods). Figure 9A shows this map of peak position discriminability for a representative subject. Figure 9B shows this region of peak discriminability superimposed on the map of the subject's positive and negative BOLD. Notice that the regions of peak discriminability (the clusters of voxels, circled with a white dashed line, that display the steepest position discrimination slope, as in Fig. 7) always occur near the subject's peak positive and negative BOLD responses. More importantly, there is little or no discriminability of object position in the anterior regions of the visual cortex. Therefore, the regions of the visual cortex that are far from the representations of the stimuli do not carry precise information about the positions of the stimuli, just as we would expect.

## Discussion

The results of the experiment here demonstrate that the negative BOLD response in visual areas V1 through V4 carries surprisingly precise information about object position. We found that as the positions of flickering Gabor stimuli were incrementally shifted, the spatial correlation in the pattern of activity decreased. The slope of this decrease was an indicator of the selectivity for object position. Surprisingly, this position selectivity was nearly as precise in the regions that displayed a negative BOLD response as in those that displayed a positive BOLD response; regions that display either a positive *or* a negative BOLD response discriminated objects separated by 0.19 deg. Stimulus-specific processing therefore occurs in areas that display a strong negative BOLD response, indicating that the negative BOLD response may be very important for understanding the mechanism of visual localization.

Although our results indicate that patterns of negative BOLD responses can be highly stimulus-selective, the physiological origin of the negative BOLD signal remains unclear. Hemodynamic explanations (“blood stealing” or “blood sharing”) posit that decreases in blood flow result from changes in the need for vascular resources elsewhere in the brain, without a necessary decrease in neuronal activity in the negative regions. In “blood stealing,” negative BOLD results from reduced local blood pressure arising from nearby capillary dilation caused by a positive BOLD response; the active regions automatically steal vascular resources from nearby inactive regions [18], [20]. In the “blood sharing” explanation, a neural system for controlling blood flow actively directs vascular resources to nearby or remote regions [6]. The results of Shmuel, et al., (2002) reveal a strong correlation in the amplitude and time course of BOLD activity between a positive stimulus-related region and surrounding negative regions. Although these authors reject “blood stealing” as the main explanation for negative BOLD, their results suggest that there is a link and perhaps a spatial dependency between the negative and positive BOLD responses in adjacent regions of the cortex. Other support for a hemodynamic origin of negative BOLD activity comes from two studies that demonstrate that negative BOLD can occur without a concomitant decrease in neuronal activity [21], [22].

None of these vascular explanations of the negative BOLD signal predict our results. It is conceivable that different patterns of positive BOLD activity could uniquely steal blood from the surrounding regions, creating patterns of negative BOLD similar to ours. However, we reject this hypothesis because of the precision of our results. All of our flickering Gabor stimuli were nearly superimposed. Further, because the patterns of positive BOLD share the same surrounding vasculature, vascular demands are spread diffusely in the surrounding regions and should not produce the very specific changes in negative BOLD activity we found. Likewise, it is difficult to see why a long-range “blood sharing” mechanism would need such specific spatial selectivity in its diversions of vascular resources. Furthermore, recent findings that vascular explanations play a limited role in negative BOLD activity in visual cortex [6], [18], [23] required us to search elsewhere for an explanation of our results.

Recent research has suggested that hemodynamic explanations are incomplete, and that the negative BOLD signal depends primarily on a decrease in neuronal activity (much as positive BOLD reflects an increase in neuronal activity). Smith and colleagues [6] presented visual stimuli to one hemisphere of the visual cortex and found sustained negative BOLD in the opposite hemisphere; since the two hemispheres have largely independent blood supplies, local blood stealing cannot explain the negative BOLD. Shmuel and colleagues [23] demonstrated that negative BOLD is correlated with decreased neuronal activity by simultaneously measuring electrical recordings and fMRI activity of visual cortex. They also show that the local decrease in neuronal activity is better than adjacent positive BOLD activity at predicting the spatial and temporal pattern of negative BOLD. Nevertheless, most research that primarily supports a neuronal explanation still allows for a hemodynamic contribution to negative BOLD [6], [18], [23].

If neuronal suppression primarily underlies the negative BOLD response, what is the purpose of this suppression? The most common explanation for this activity is based on attentional processes. It is well known that when attention is directed to a location, information processing is facilitated at that location and suppressed at nonattended locations [24], [25], [26], [27], [28]. In terms of neuronal activity, many studies have demonstrated an increase in neuronal activity at the attended region, even in the absence of visual stimulation, and a reduction in neuronal and BOLD activity at unattended regions [7], [9], [25], [29], [30], [31], [32], [33], [34], [35]. Although there are many variations on attentional explanations with respect to the BOLD response [5], in general it is thought that attention improves accuracy by increasing the neuronal activity at the attended site and decreasing activity in unattended regions, thereby boosting the relevant signals and mitigating contributions of noise. It is worth noting that this explanation incorporates the negative BOLD signal as an active contributor to the attentional process; accuracy is increased by the inhibition of noise signals.

Attentional explanations of the negative BOLD signal provide a better account of our results than the hemodynamic explanations above because they propose a meaningful role for neuronal suppression (and the resulting negative BOLD). However, these explanations do not entirely predict our results because they maintain that attentional suppression occurs broadly, over a wide region of visual space [5], [7], [9], [34], [35]. The corresponding negative BOLD activity either occurs at all other retinotopic locations [5] or demonstrates relatively weak spatial specificity [7], [9], [34], [35]. No studies have reported the extremely high spatial selectivity of negative BOLD, nor have they theorized a need for such specificity. Our results, however, showed that the negative BOLD response surrounded the positive BOLD response to the target object in a systematic and precise manner. This sort of center-surround response [7], [36] could serve to improve the resolution of position coding [37].

One of the strengths of the attentional mechanism above is that it also explains why we found such precise discrimination in later visual areas (e.g., V4). Previous research has established that retinotopic organization is most precise in V1 and less so in later visual areas such as V3 and V4 [38], [39], [40], [41], [42], [43]. It is therefore surprising that in our study the position discrimination slopes for positive BOLD activity in later visual areas are almost as high as the slope for V1 (Fig. 7C). However, several studies have found that attention has greater modulatory effects in visual areas beyond V1 and V2 (such as V4, [31], [44]), so it is possible that the sustained attention inherent in our task increased the discriminability in these regions. That attention improved the sensitivity of these regions, nearly equaling the ability of V1, is evidence of the potential importance of attentional mechanisms in object localization.

The implication of our study for fMRI research on attention and perception is that what is suppressed may be as important as what is activated. Other studies [45], [46] have demonstrated that neuronal suppression (and the concomitant negative BOLD) may be a main component of a top-down attentional system, and that the failure to suppress information may be central to selective performance deficits. Although these studies do not involve spatial attention, they emphasize the potential importance of neuronal suppression for cognitive functions. Given the precision of the negative BOLD responses we found, our study raises the possibility that the suppressive annulus surrounding an attended area may contribute substantially to the resulting percept. Consistent with this suggestion, Figure 9 demonstrated that the peak discrimination of object position occurred near or between the regions of peak positive and negative BOLD response—near the edges of the object. This makes sense, as the mechanism responsible for perceptual localization may depend on the precision with which the edges of the object are coded [37], [47], [48]. Our study indicates that the negative BOLD signal, far from being an unrelated artifact of perceptual processing, is highly informative of the spatial characteristics of visual stimuli.

## Materials and Methods

### Stimuli

Stimuli in the main experiment consisted of four Gabor patterns (sine wave luminance modulations with a Gaussian contrast envelope, Fig. 1). The peak contrast of each Gabor (87% Michelson) was always centered at 9.05 deg eccentricity. The spatial frequency of the luminance sine wave was 0.38 cyc/deg. The Gaussian contrast envelope of each Gabor was defined as 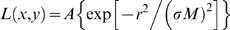, where *A* is the peak contrast amplitude, *r* is the distance of *(x,y)* from the center of the Gaussian, *σ* is standard deviation, and *M* is the maximum radius. Each Gabor was flickered in counterphase at 7.5 Hz. The phase of each Gabor was randomized on each trial.

There were six conditions in the experiment. One of these conditions was a fixation baseline (nothing was visible but the fixation point). In the other five conditions, the Gabor stimuli were skewed either toward or away from the fixation point by varying amounts. In one of these conditions, the Gabors had a symmetrical Gaussian contrast envelope (Fig. 1C) with a standard deviation of 1.66 degrees. In the other four conditions (Fig. 1A, B, D, E), the contrast envelope was skewed toward or away from fixation by an additional 0.19 or 0.38 degrees, for a total of 5 test conditions (0.38, 0.19, 0, −0.19, and −0.38 deg skew in the standard deviation of the contrast envelope; negative values indicate a skew toward the fovea). To achieve an asymmetrical skew in each Gabor, the Gabor was divided in half (one half closer to fixation and one half more eccentric), and the standard deviation of the Gaussian envelope was independently determined for the two halves of the Gabor (similar to the method of Whitaker and colleagues [49], [50]). That is, the half of the Gaussian envelope closer to fixation had a different standard deviation than the half further away from fixation. We have chosen to express the Gabor positions in terms of the standard deviation of their contrast envelopes, but one could express Gabor location in terms of stimulus centroid [51], in which case the five Gabor centroids in Fig. 1 were 8.5, 8.75, 9.04, 9.32, and 9.6 deg. Using either standard deviation of contrast envelope or stimulus centroid to express the Gabor positions does not change the pattern of our results, or the significance tests. The peak contrast in the position of the Gabors remained fixed in all conditions at 9.05 deg. Skewing the contrast envelope rather than shifting the overall position of the Gabors is a better method of isolating visual mechanisms that code object positions [49], [50], [51], [52].

In each functional imaging run, the six conditions were randomly interleaved in 36 ten-second blocks (360 sec runs). Each subject participated in a minimum of five functional runs (except for subject DB, who participated in four runs). Subjects maintained fixation at a central point (0.39 deg diameter) throughout the entire experiment.

In separate localizer runs, we presented flickering bowtie stimuli to identify the borders of visual areas V1 through V4. The bowties consisted of radial sine wave patterns that were 11.79 deg radius and subtended an arc of 8.16 deg. The bowties flickered in counterphase at 7.5 Hz. There were three conditions in these retinotopy runs; in two conditions the bowties were centered on the vertical or horizontal meridians, and the third condition was a fixation baseline. Conditions were randomized in 36 ten-second blocks.

### Task and attention control in the main experiment

At a randomly chosen time during the first 8 seconds of each 10 second block, a small texture pattern (either circular or radial grating, chosen randomly; 1.09 degrees diameter) was flashed for 500 ms superimposed on one of the 4 Gabors (chosen randomly) at an eccentricity of 9.05 deg (center to center) from fixation. The flashed texture was presented during every condition, including the fixation baseline. During the last 2 seconds of each 10 second block, a second flashed texture was presented superimposed on the Gabors for 500 ms and a white annulus (0.98 deg diameter) was presented continuously around the fixation point, indicating that subjects should make a response. The second textured flash matched the first one with a probability of 50%.

Subjects were instructed to maintain fixation at all times and make two judgments. First, subjects discriminated the eccentricity of the Gabors (the degree of skew in their envelopes) in a 5 alternative-forced-choice task (by pressing one of five keys on a button box). Subjects also needed to continuously monitor the 4 Gabors for the texture flashes. If the two textures matched, subjects pressed the position selection button once; if the two flashes did not match in texture, subjects pressed the position selection button twice. For example, if the Gabors appeared to be skewed to the most eccentric position and the two textured flashes did not match, the subject would press the appropriate key twice. This task was designed to focus subjects' attention on the surrounding Gabors. Notice that to successfully complete the task, subjects needed to maintain their attention on the cued locations throughout the length of each block.

A small textured annulus (either circular or radial grating, chosen randomly; 0.98 deg diameter) was flashed (500 ms) surrounding the fixation point at least 7 times but no more than 11 times, and one texture was always presented once more than the other. These flashes were uncorrelated with the textured flash superimposed on the Gabors and were therefore uninformative. Subjects were instructed to ignore the flashes at the fixation point and to attend entirely at the position of the Gabors.

### fMRI data collection and analysis

Seven subjects participated in the experiment. Scanning protocols were approved by the University of California, Davis, Human Subject Review Board. Imaging was conducted on a 3-Tesla Siemens TRIO scanner located at the UC Davis Imaging Research Center. Each participant's head was rested in a Siemens eight-channel phased-array head coil. Braces and padding on the side and forehead of the participant restricted head motion and provided feedback to the subject about any potential body movements. Stimuli were back-projected with a Digital Projection Mercury 5000HD projector (75 Hz) onto a semi-transparent screen from outside the bore. A mirror angled at 45 deg, located 9.5 cm directly above the subject, provided a reflected view of the stimuli. Functional images were acquired with a gradient-recalled echo EPI sequence. Whole-brain structural images were collected with a high resolution (1 mm^3^) Turbo Spin Echo scan that was used to align functional images. The acquisition parameters were: TR = 2000 ms, TE = 26 ms, FA = 90 deg, FOV = 22×22 cm^2^, voxel size = 1.528×1.528×2.5 mm^3^, 20 slices per volume. The imaging volume was parallel to and centered on the calcarine sulcus, covering the occipital lobe (Fig. 3).

All preprocessing, including linear trend removal and 3D motion correction, as well as GLM analyses, were conducted with Brain Voyager QX (Brain Innovation B.V., Maastricht, The Netherlands). The images were not spatially smoothed and no mean image normalization was applied. A correction for serial correlations (removal of first-order autocorrelation) was used prior to all GLM analyses. Each subject's high resolution anatomical image was transformed to Talaraich coordinates, and the data for each functional run was individually aligned to the subject's Talairach-transformed anatomical image. Performing individual alignments for each functional run, within each subject, mitigated any effect of subject movement between functional runs.

We performed general linear model (GLM) analyses on the data in the retinotopy runs to define visual areas V1 through V4. Bowties along the vertical meridians were contrasted with those on the horizontal meridians, which yielded a striated map of activity across early visual areas; the boundaries of each visual area V1 through V4 were defined by the mirror reversals in the representations of the horizontal and vertical meridians.

In the main experiment, for each functional run, a GLM was fit to the data with five predictors (corresponding to the five Gabor positions). These five Gabors were separately contrasted against a sixth predictor (the fixation baseline) to discover areas of peak activity for each Gabor position. Separate activation maps based on the GLM were created for each of the five Gabor positions. In these three-dimensional maps, every voxel had a statistical value associated with it (there was no threshold—each voxel had a t value, though many were very close to zero).

In separate GLM analyses, we contrasted all five Gabor conditions to the fixation baseline. In addition to the substantial positive BOLD response throughout the visual cortex, this GLM also revealed large regions of negative BOLD activity. Within each visual area (V1 through V4), we defined separate regions-of-interest (ROIs) for the negative and positive BOLD response for each subject. The threshold for inclusion in the ROI was t>±5.6, P<0.05, Bonferroni corrected. However, three subjects had a weak negative BOLD response, and therefore did not display negative BOLD in every visual region at this threshold. For these subjects we dropped the threshold for inclusion in each ROI to t>±2.9, P<0.05. In a separate analysis, we constructed a single ROI of the negative BOLD activity in all visual regions. For the subjects with the lower threshold, there was no difference in the position discrimination slope between the overall ROI at the higher threshold and the overall ROI at the lower threshold.

Our goal was to correlate the spatial pattern of activity produced by each of the Gabor stimulus conditions with the others, within each ROI. All correlational analyses (c.f., [53]) were conducted with Matlab 7.1 (The Mathworks, Natick, Massachusetts). Within each ROI, we cross-correlated the volumetric statistical maps (statistical t values) produced by each of the five Gabor positions (for a total of ten correlations). We converted these r values to Fisher z scores because equal distances between these scores are equally probable [19], and Fisher z scores can be directly compared, unlike r values. The highest z-score was identified within each ROI, and the differences between the highest z-score and all 10 z-scores was computed, for a total of 10 normalized Fisher z-score differences. Larger Fisher z score differences indicate a lower spatial correlation between the two stimulus conditions being compared.

The 5 positions of the Gabor stimuli (i.e., the five degrees of skew in the standard deviation of the Gabors' contrast envelopes) were 0.38, 0.19, 0, −0.19, and −0.38 deg. Thus, the greatest distance between any two positions was 0.77 (between a skew of 0.38 deg outward and a skew of 0.38 deg inward), and the slightest difference in the position of any two conditions was 0.19 deg. The differences in each pair of Fisher z scores (above) were plotted as a function of the difference in the skew between each pair of conditions, and the slope of a linear regression fit to the data was computed.

The slope of the linear regression is a measure of the selected ROI's ability to discriminate position shifts (i.e., Gabor skew). Note that if the pattern of activity in the ROI showed no selectivity for object position, the slope of the linear regression should be zero. However, if the ROI can discriminate object position, then the spatial correlation of activity produced by two Gabor stimuli should be higher when the two Gabors are nearer to each other. Therefore, the linear regression slope within a given ROI is an indicator of that region's ability to discriminate changes in position.

In a separate analysis, we defined every possible 5×5×5 cube of voxels in the entire brain, creating thousands of ROIs. Because many of these ROIs overlapped, each voxel in the brain was covered by 125 ROIs. We performed the above analysis to obtain a position discrimination slope value for each ROI. Because any particular voxel was covered by 125 ROIs, we assigned the average slope of those 125 ROIs to that particular voxel. Each voxel therefore represents the average position discrimination ability of the overlapping ROIs that surround it. With these average position discrimination values, we created a new map revealing clusters of voxels that are best able to discriminate the Gabor positions.
